# Rapid post-eruptive maturation of porcine enamel

**DOI:** 10.3389/fphys.2023.1099645

**Published:** 2023-02-16

**Authors:** Baptiste Depalle, Hakan Karaaslan, Nicolas Obtel, Ana Gil-Bona, Maren Teichmann, Gabrielle Mascarin, Megan Pugach-Gordon, Felicitas B. Bidlack

**Affiliations:** ^1^ The Forsyth Institute, Cambridge, MA, United States; ^2^ Department of Developmental Biology, Harvard School of Dental Medicine, Boston, MA, United States; ^3^ École Normale Supérieure, Paris Sciences et Lettres Research University, Paris, France

**Keywords:** enamel, maturation, porcine (pig) model, mineralization, tooth formation, posteruptive hardening

## Abstract

The teeth of humans and pigs are similar in size, shape, and enamel thickness. While the formation of human primary incisor crowns takes about 8 months, domestic pigs form their teeth within a much shorter time. Piglets are born after 115 days of gestation with some of their teeth erupted that must after weaning meet the mechanical demands of their omnivorous diet without failure. We asked whether this short mineralization time before tooth eruption is combined with a post-eruptive mineralization process, how fast this process occurs, and how much the enamel hardens after eruption. To address this question, we investigated the properties of porcine teeth at two, four, and sixteen weeks after birth (N = 3 animals per time point) through analyses of composition, microstructure, and microhardness. We collected data at three standardized horizontal planes across the tooth crown to determine the change of properties throughout the enamel thickness and in relation to soft tissue eruption. Our findings indicate that porcine teeth erupt hypomineralized compared to healthy human enamel and reach a hardness that is similar to healthy human enamel within less than 4 weeks.

## Introduction

Porcine teeth resemble human teeth in terms of size, morphology, and enamel thickness (Robinson et al., 1987; 1988b). One major difference however is that pig teeth develop four and a half times faster than human permanent teeth (Schour L and Massler M, 1941; Tonge and McCance, 1973; Birch and Dean, 2014). As a result, pig enamel is far softer and more porous than healthy human enamel at the time of tooth eruption (Kirkham et al., 1988). However, the stunning tooth formation speed in pigs raises two questions. First, how quickly can enamel be formed using a cellular mechanism? Second, how fast and efficient is the cell-free enamel hardening after eruption? While the evolution of mineral crystals prior to tooth eruption has been characterized in depth (Kallistova et al., 2017), little is known about the development of pig enamel once it reaches the oral cavity.

There is evidence that, at the time of tooth eruption, enamel is not fully mineralized and that its maturation and hardening continue in the oral cavity (Yonan et al., 1966; Fejerskov et al., 1984; Wöltgens et al., 1990; Palti et al., 2008). However, the speed of this natural process and how deep into the enamel layer the hardening occurs has not been described. The level of enamel mineralization of permanent porcine teeth is known to reach only 85% by weight (60% by volume) at the end of cell mediated maturation, just at the time of eruption and when the tooth reaches the oral cavity (Robinson et al., 1988b; Kirkham et al., 1988). This mineral content is significantly lower than healthy human enamel where mineral content is higher than 95% by weight prior to eruption (Kirkham et al., 1988). In fact, porcine enamel is with its high protein content in erupted teeth similar to human hypomineralized enamel as described for idiopathic demarcated opacities with a reported 58.8% mineral by volume (Mahoney et al., 2004; Crombie et al., 2013; Elfrink et al., 2013).

**FIGURE 1:**
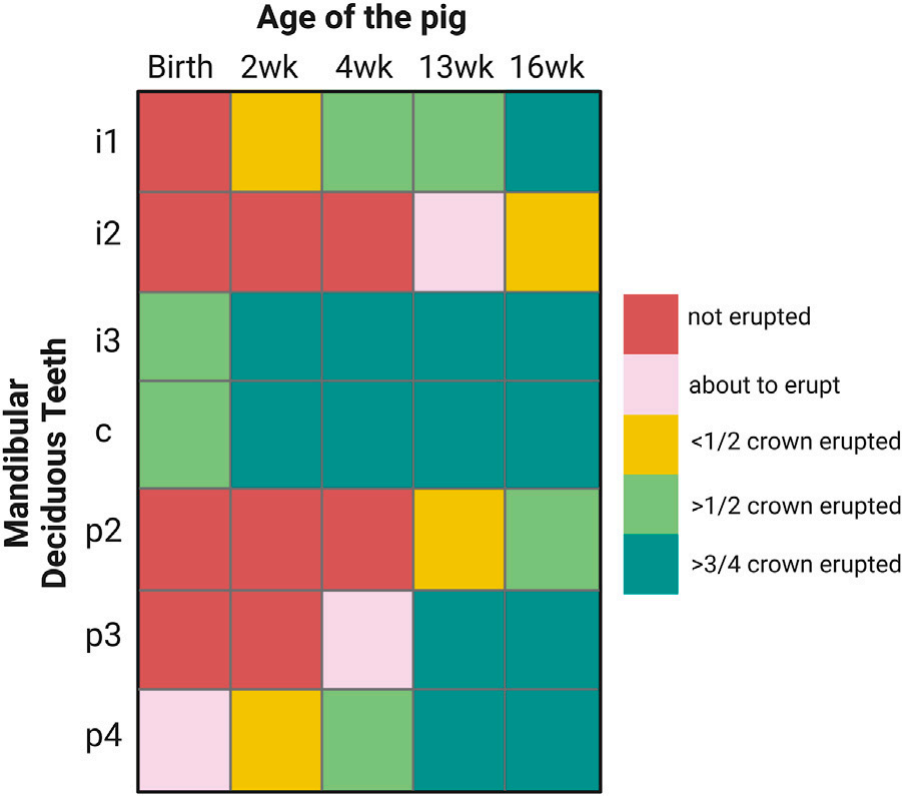
Eruption sequence of 2, 4, 13 and 16-week-old pigs that are used in this study. I: incisor, c: canine, p: premolar.

Porcine enamel formation occurs so quickly in those teeth that are erupted at birth or shortly thereafter that the last formation stage of enamel maturation is not completed. Piglets are born with their mandibular canines and third incisors erupted. By two weeks after birth, less than half of the first lower incisor crown is erupted (Figure 1). In comparison, the first teeth to erupt in humans, incisors, also form “on the fast track” before birth (Mahoney, 2015). However, after at least 90 days prenatal and 30 days postnatal crown formation time, several months remain for enamel maturation before tooth eruption sometime after the baby’s first 3 months after birth (Liversidge and Molleson, 2004; Mahoney, 2015).

The goal of this study is to advance our understanding of the speed and depth of porcine enamel hardening after tooth eruption. Our data and new insights can provide guidance for strategies of hardening hypomineralized human enamel and can inform efforts of regenerating teeth.

## Materials and methods

All samples were harvested after sacrifice of Yorkshire pigs raised and weaned after 4 weeks at the animal facility of Tufts University Cummings School of Veterinary Medicine following approved IACUC regulations (protocol number G2019-72).

### Choice of teeth

The selection of teeth used for this study is based on the timing and sequence of tooth eruption in pigs. The canine and third lower incisor are erupted at birth, known as needle teeth, but get clipped upon birth and were not included in this study. Here, we focused on mandibular teeth, in particular, on the first incisor which is by 2-week of age less than half erupted (Figure 1). We also used premolars. At 2 weeks of age, the fourth premolar is less than half erupted, at 4-week, the third premolar is emerging and at 13-week, less than half of the second premolar crown is erupted (Figure 1).

### Sample harvesting and preparation

Pigs aged 2 weeks (n = 3), 4 weeks (n = 3) and 16 weeks (n = 2) were euthanized, mandibles dissected out and immediately transferred into dry ice for transport, then stored at −80°C until further processing (Table 1.). The first mandibular incisor was carefully extracted to minimize contamination. The erupted part of the teeth was cleaned with a toothbrush and PBS to remove pellicle and plaque. We used the second, third, and fourth premolars, and the first incisor from the lower left mandible of a 13-week-old pig for fluorescein application to the crown surface and measures of penetration as an approximation of porosity (Table. 1). Therefore, at the time of extraction, the time that a tooth spent in the oral cavity ranges from 5-week for the second premolar, 9-week for the third premolar and, to 11-week for the fourth premolar and first incisor.

**TABLE 1:** Age and number of animals used for each experimental procedure.

Analysis\Age of pig	2 weeks	4 weeks	13 weeks	16 weeks
microCT, SEM Microhardness (HV)	n = 3 (1 tooth/animal)	n = 3 (1 tooth/animal)		n = 3 (1 tooth/animal)
Fluorescein penetration			n = 4 (4 teeth/animal)	

### Mineral density

**FIGURE 2:**
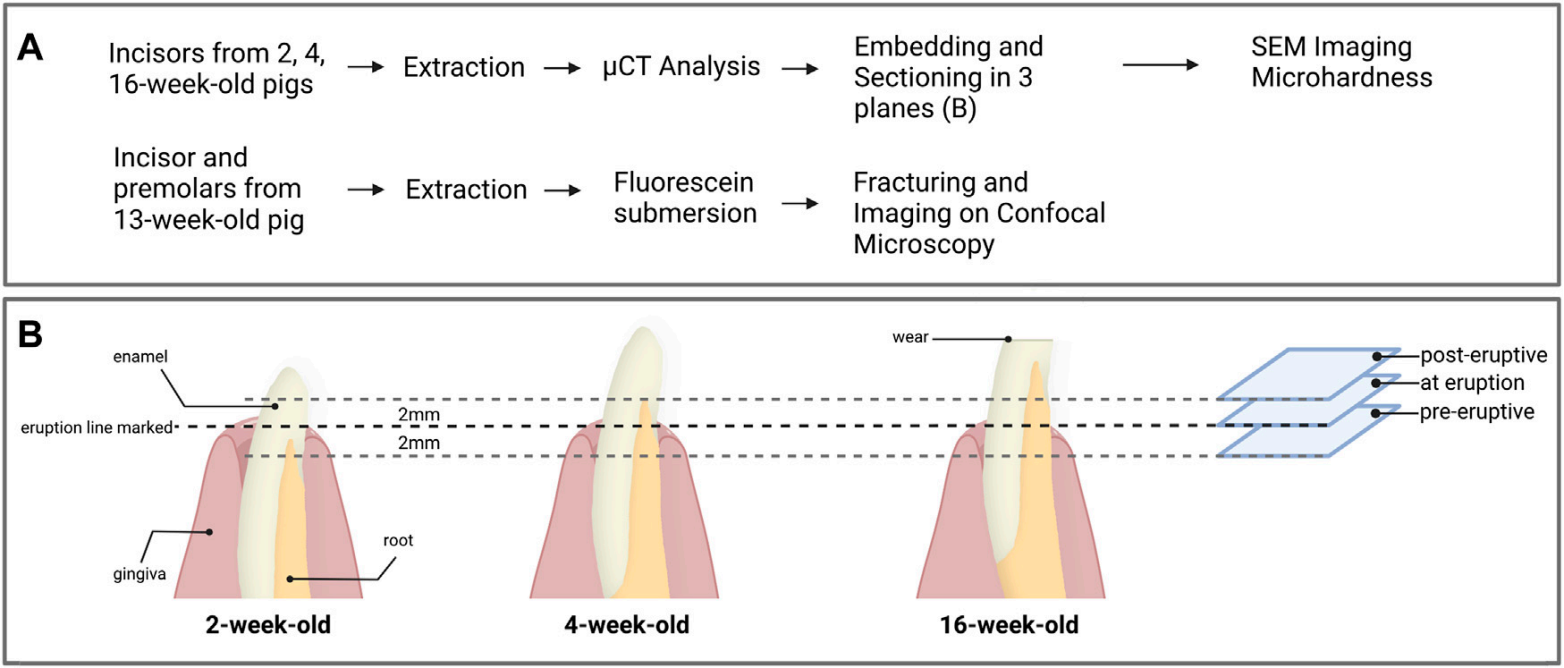
**(A)** Overview of the experimental workflow and **(B)** processing of the first incisors for each age groups. Prior to extraction, the eruption line was marked on the incisors from 2, 4 and 16-week-old pigs. After extraction and microCT analysis, the samples were embedded and three transverse sections were prepared 2 mm below the gingival emergence, at the level of gingival emergence, and 2 mm above the gingival eruption line. Each section was used for the acquisition of data from pre-eruptive, at eruption and post-eruptive enamel for a given tooth.

After extraction, incisor teeth from 2, 4 and 16-week-old pigs were analyzed by microcomputed tomography microCT on a Scanco µCT40 scanner (Scanco Medical, Switzerland) with a 6 μm voxel resolution. The 3D reconstruction of the samples was reoriented to align the long axis of the tooth with the vertical axis. The enamel density varies significantly from cuspal to cervical margin and includes densities that are similar, or lower compared to dentin, resulting in an overlap in the histogram. Therefore, classic segmentation methods like intensity threshold are not applicable here. Thus, the enamel was segmented with Deep Learning in Dragonfly software (Dragonfly 4.2, The Objects). We used a UNet segmentation model with a categorical cross-entropy loss function and the Adadelta optimizer. The training was performed on a representative subset of images from the microCT tomograms and manually segmented. After segmentation, the average mineral density of enamel was computed in FIJI (version 1.53c) (Schindelin et al., 2009) from the root to the crown of the tooth. The location of the plane of gingival emergence was confirmed by matching images obtained of the cross-section of the tooth in SEM with the images from the tomogram (Figure 2). Additionally, the mineral density of enamel near the crown surface was evaluated using a mask corresponding to the outer 30 µm of enamel. For each sample, the mineral density was smoothed by computing the moving average over 5-data points. The average and standard deviation of the smoothed data from three samples is reported thereafter. As the enamel near the cervical margin is close to the microCT voxel size, the data are susceptible to artifact such as partial volume effects and segmentation errors. This can lead to noisy data and unreliable results. To minimize these effects, a minimum area of 0.02 mm^2^ has been used as a threshold before computing enamel mineral density.

### Sample preparation for analyses of enamel microstructure and microhardness

Following the micro-CT analysis, incisor teeth were embedded in London White resin (LR white) and sectioned transversally with a low-speed saw using a diamond blade (Isomet, Buehler). The teeth were oriented in relation to the line of gingival emergence, which was measured from the cusp of the tooth before extraction and confirmed by detection of a distinct change in enamel color. Each tooth was sectioned horizontally at the level of gingival emergence, then additional sections were placed 2 mm below and 2 mm above the eruption line (Figure 2B). At each section plane, we used the exposed face of the incisal portion of the tooth for SEM data acquisitions and the mirroring face of the cervical portion of the sample for microhardness testing. All section surfaces were polished through a series of polishing papers to 0.3 µm.

### Analyses of enamel microstructure by scanning electron microscopy (SEM)

Polished sections were etched for 10 s with 0.1 M phosphoric acid, rinsed with deionized water for 1 min, air dried, and gold coated. Imaging was performed with a Zeiss Ultra 55 SEM at 3 kV and a 5 mm working distance. The imaged area was selected at the center of the buccal part of the tooth, far from any major defect at the surface of the enamel.

### Microhardness testing

**FIGURE 3:**
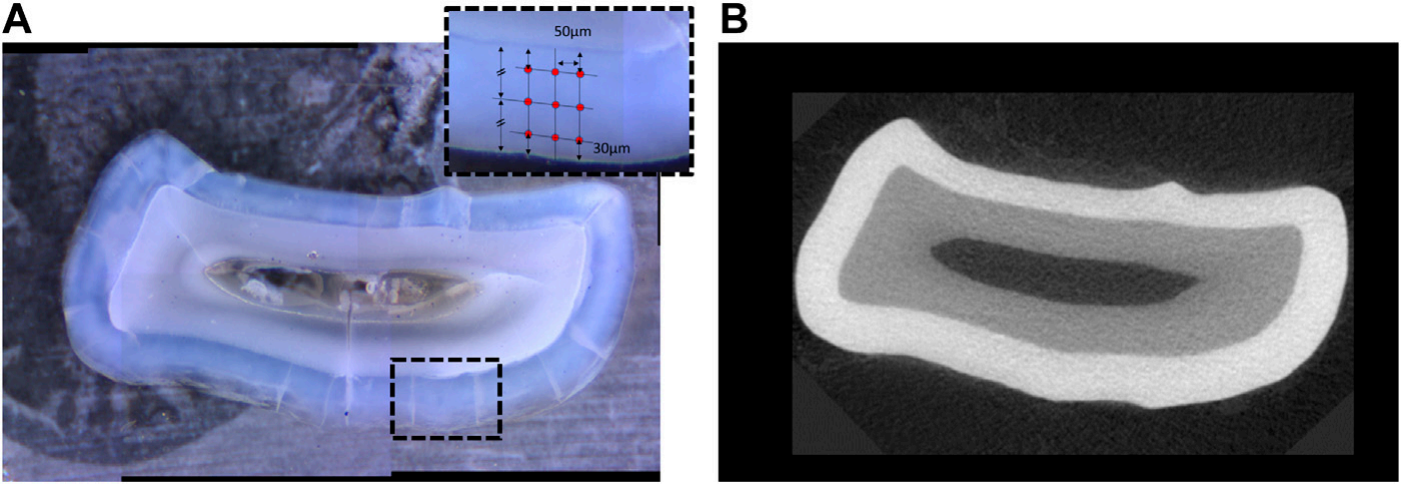
**(A)** Transverse section of a mandibular incisor at the level of gingival emergence, with inset in panel representing the location of the indents of the micro-indenter in one of the four quadrants of the cross-section. **(B)** Corresponding image of the same tooth in a virtual section at the level of gingival emergence from the microCT tomogram (right) showing mineral density.

The polished samples were mounted with cyanoacrylate on a metallic disk and data acquired on a M400 HI Vickers testing machine (Leco, St. Joseph, MI) with a load of 25 g and a 5 s dwell time. Each tooth section was tested at two locations on the buccal and two locations on the lingual side with nine indents per site comprising three each in the inner enamel, near the dentin-enamel junction, three in the mid-enamel and three in the outer enamel, near the surface of the tooth (Figure 3).

### Porosity indicated by fluorescein penetration

The second, third, and fourth premolars and the first incisor (n = 1 per tooth type) from a 13-week-old pig were ultrasonicated for 3 min to clean the enamel surface and the samples were submerged into freshly prepared 1 mM fluorescein for 45 min at 4°C. The samples were then rinsed in deionized water and fractured bucco-lingually in the midplane of the crown, glued on a glass slide with epoxy resin. A z-stack of images of the cross-section of enamel was taken by confocal microscopy on a Zeiss LSM 780 Confocal Microscope (Zeiss, Germany). A qualitative assessment of the penetration depth of fluorescein from the crown surface was assessed in maximum intensity projections.

### Statistical analysis

Statistical analysis was performed using Prism 9 (Graphpad Software Inc., San Diego, CA). The microhardness values were analyzed using two-way ANOVA and multiple comparisons were performed using Tukey’s test, where α = 0.05.

## Results

### Mineral density increases drastically between 2 and 4 weeks after birth in the crown surface

**FIGURE 4:**
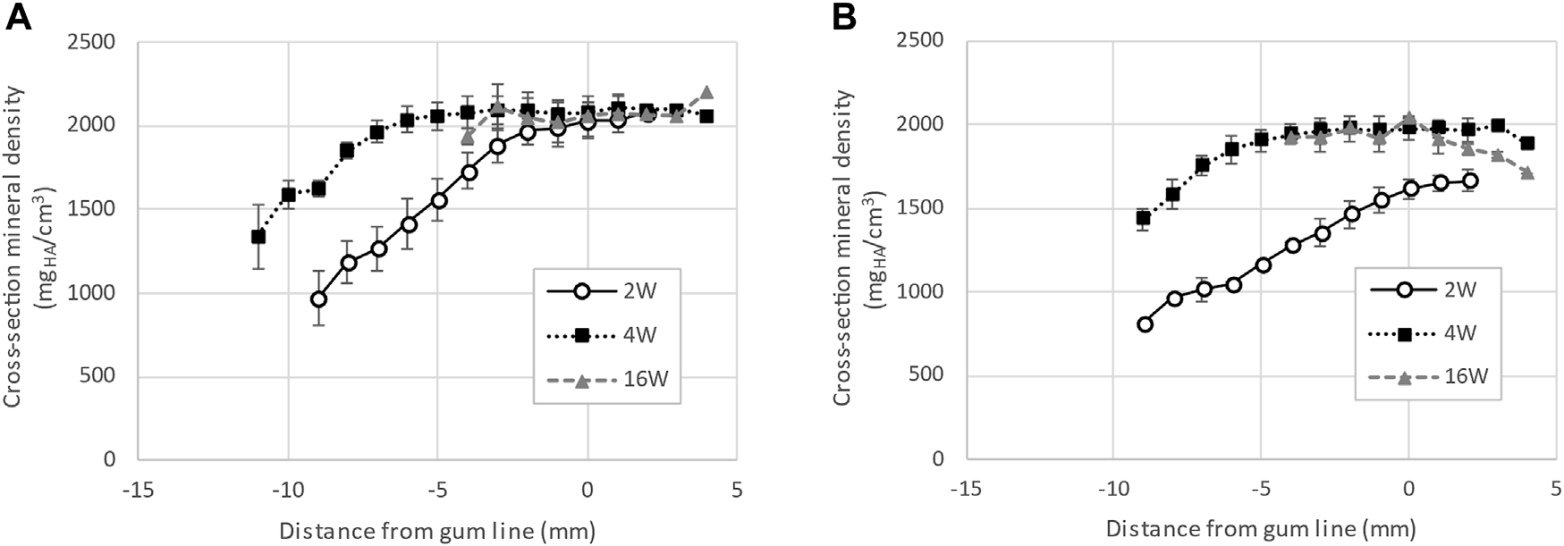
Progression of enamel mineral density for incisors, extracted from pigs aged 2, 4 and 16 weeks. Panel **(A)** shows the average density measured along the cross-section of the tooth while panel **(B)** shows the average density of the 30 µm near the surface of the tooth. The data indicates that enamel near the surface of first incisors is not fully mineralized at eruption in 2-week-old pigs.

The comparison of enamel mineral density between teeth of the same type, namely first incisors of the primary dentition, harvested from piglets at different ages shows a marked difference at the crown surface. Specifically, measures of mineral density derived from a series of virtual sections comprising 3 mm of the erupted crown height from the level of gingival emergence shows a clear progression of the mineral density as see in Figure 4. The enamel mineral density within the 30 µm surface layer of the erupted porcine incisor crown was on average 1644 ± 62 mg_HA_/cm^3^ in 2-week-old animals. These values were measured between the level of gingival eruption and 3 mm above and increased in animals 4 weeks of age, to 1977 ± 68 mg_HA_/cm^3^. No further increase was seen in the 16-week-old pigs, where the enamel mineral density at the incisor surface was 1959 ± 90 mg_HA_/cm^3^.

### Between 2 and 4 weeks after birth, the crown surface porosity decreases

**FIGURE 5:**
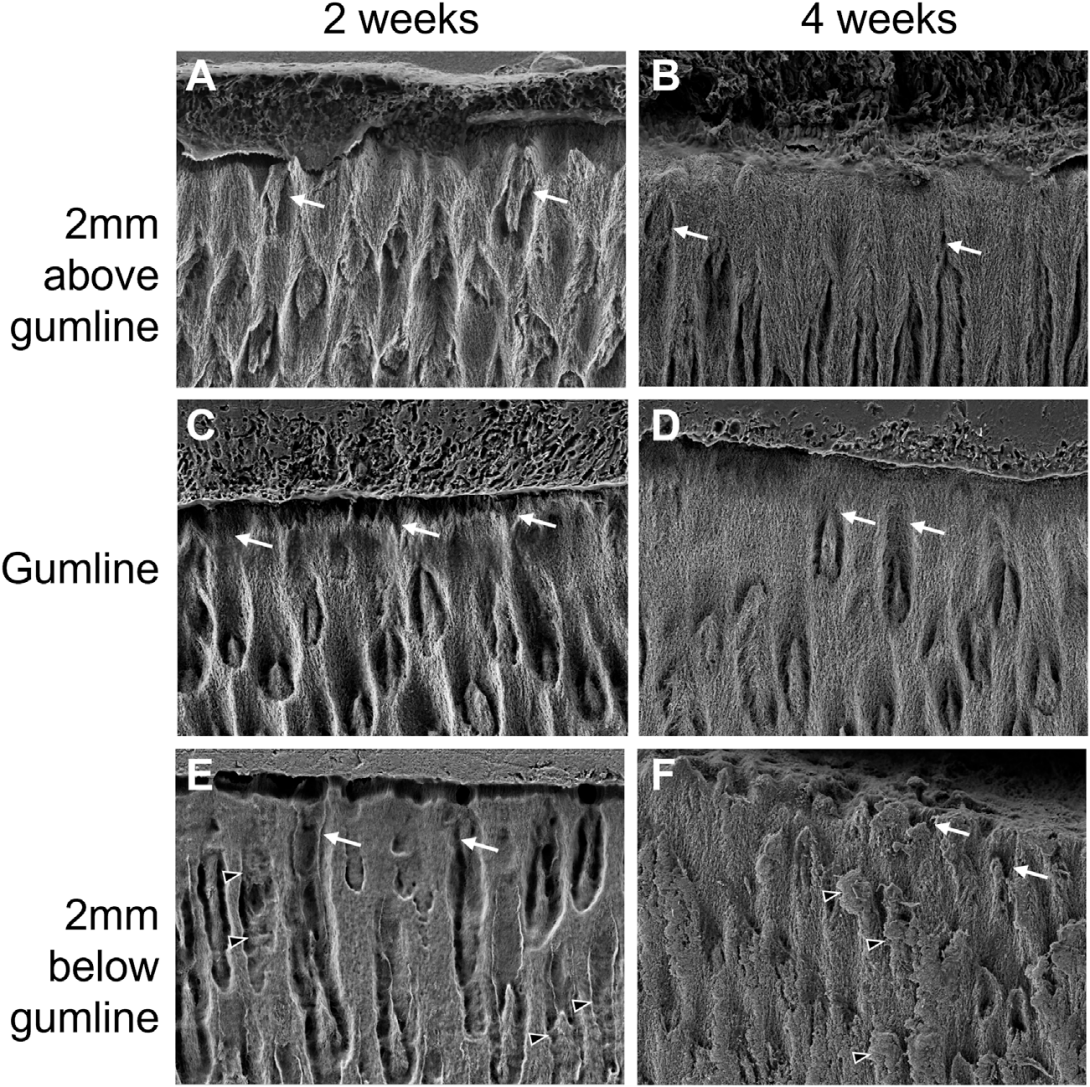
Progression of enamel surface micro-structure with eruption and with age in the first lower left incisor of pigs aged 2 weeks **(A, C, E)** and 4 weeks **(B, D, F)**. **(A, B)** show surface enamel 2 mm above the gingival eruption level, referred to as gumline, seen in **(C, D)**, with surface enamel 2 mm below the gumline in **(E, F)**. Black arrowheads: proteins; white arrows: porosity.

The enamel in the first incisor from 2-week-old pigs presents a large amount of porosity (Figures 5A,C,E). Below the gumline, large channels can be observed, running from the surface of the tooth towards the dentin-enamel junction (white arrows in Figure 5). A significant amount of protein matrix can be observed along these channels as indicated by the absence of features such as sharp edges and clear shape that are characteristic for the distinct morphology of enamel crystallites (black arrowheads in Figure 5A,E,F). While the size of the channels progressively decreases in diameter when moving towards the crown of the tooth, they are still present at gumline and 2 mm above gumline. This suggests a structural porosity that would allow for exchanges with the outside environment to favor mineralization. The surface of the enamel presents some roughness which indicates that the enamel is not completely sealed, even after eruption. At 4 weeks, the incisors still present some evidence of channels running perpendicular to the surface of the tooth (Figures 5B,D,F). However, these channels appear partially sealed at the gumline and 2 mm above gumline (Figures 5D,F). The tooth surface is more mineralized, and the roughness is significantly decreased when compared to the teeth taken from younger animals.

**FIGURE 6:**
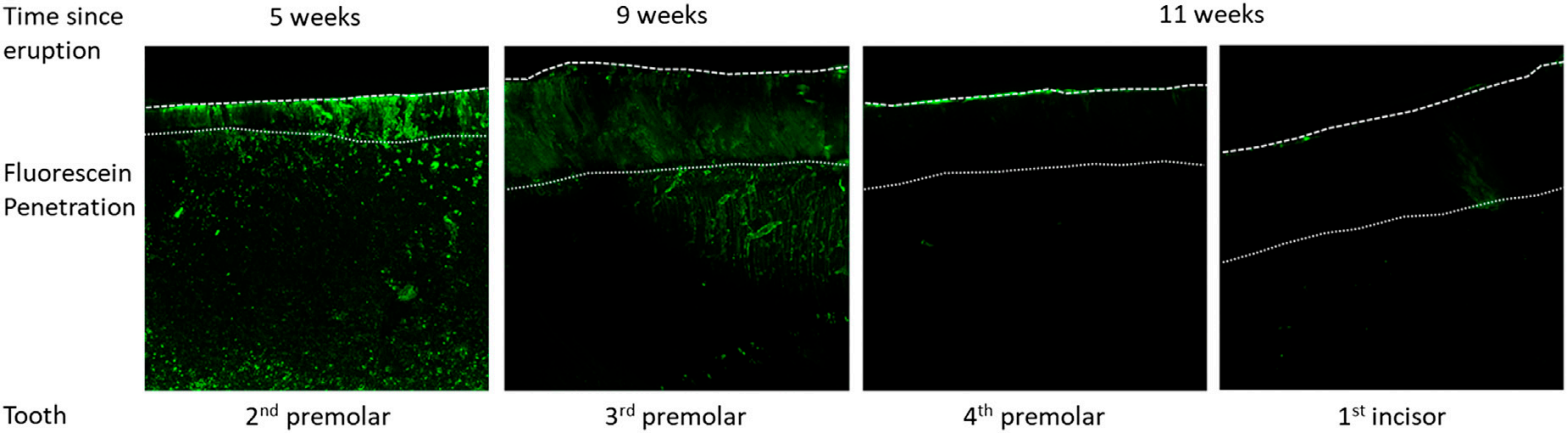
Fluorescein penetration after application on the surface of the tooth crown, followed by cleaving the tooth to expose the fracture surface that is imaged by confocal laser scanning microscopy to detect fluorescein distribution within enamel as a function of time post-eruption.

The porosity of erupted enamel, as evaluated by the penetration of fluorescein in the tissue, significantly increases as a function of the time spent in the oral cavity (Figure 6). The second premolar, which is the last tooth to erupt, is still highly porous after 5 weeks post-eruption. At 9 weeks post-eruption, the third premolar still presents some porosity across the full cross-section of enamel. At 11 weeks post-eruption, teeth appear almost impermeable to fluorescein. Taken together, our results indicate an initially high and progressively decreasing porosity and penetration of fluorescein, suggesting that enamel permeability and exchange processes with the oral cavity change after eruption.

### Fast post-eruptive hardening is the shortcut to produce functional teeth by the time of weaning

In the first incisors of 2-week-old pigs, the cross-sectional mineral density of enamel increases linearly along the length of the tooth from the cervical margin to the gum line where it reaches a plateau at a density of 2018 ± 92 mg_HA_/cm^3^. In 4-week-old pigs, the fast progress of enamel maturation occurs below the gum line. The mineral density reaches a plateau of mature enamel with a density of 2083 ± 77 mg_HA_/cm^3^ at about 5 mm below the gingival eruption line and does not further mature after. At 16 weeks after birth, the incisor crown is fully formed and has reached the occlusal plane, and the cross-sectional mineral density, including the crown surface, is mostly constant along the entire length of the tooth with a value of 2063 ± 118 mg_HA_/cm^3^.

The crown of incisors from 16-week-old animals are significantly worn which leads to a final erupted length comparable to the same tooth taken from 4-week-old pigs. Although the enamel near the occlusal surface in 16-week-old pigs has a slightly decreased mineral density, there is no apparent difference in the plateau of the average cross-sectional mineral density of enamel in the erupted part of incisors form pigs aged 2-, 4- and 16 weeks. This is in contrast to the changes in mineral density at the crown surface.

### Fast crown formation for the first teeth to erupt, fast pre-eruptive mineralization for later erupting teeth

We measured the progression of mineralization in incisors to evaluate differences between age groups. It is experimentally particularly challenging to measure the rate of mineralization over time. Therefore, we evaluated the mineralization rate by comparing the progression of enamel mineral density, ρ , along the length of the tooth. The rationale is that, for any given section taken at a position *x* along the cervical-occlusal direction of the tooth at a time *t*, its mineral density at time *t+1* is equal to the mineral density of the section at a position x+δx at time *t*. Therefore, instead of assessing the mineralization rate of enamel over time, we computed the progression in mineral density along the long axis of the tooth:

$$∆\rho =\frac{\rho \left(x+\delta x\right)-\rho \left(x\right)}{\rho \left(x\right)}$$

**FIGURE 7:**
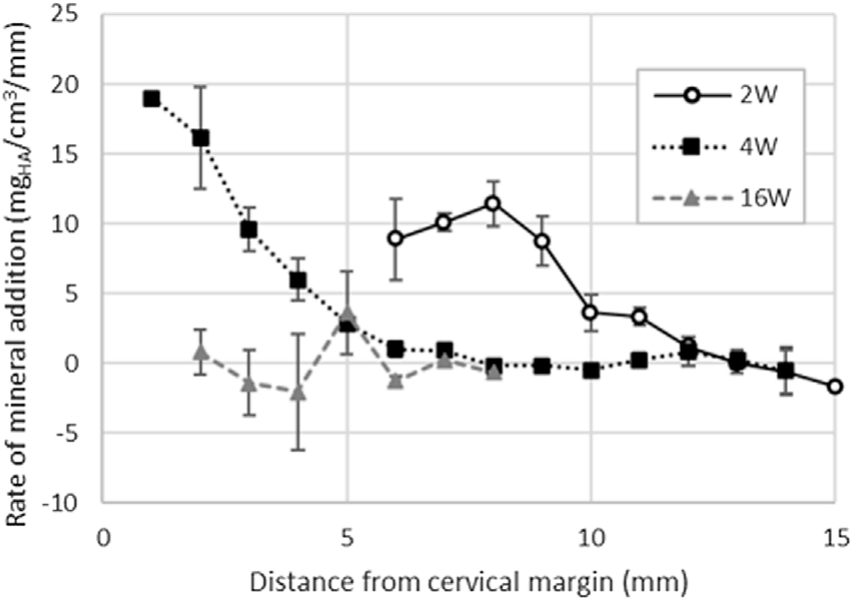
Enamel rate of mineral addition along the cervical-occlusal direction of the tooth computed for first incisors extracted from pigs aged 2, 4 and 16-week. The results indicate that the mineral addition reaches a maximum around 8 mm form the cervical margin in 2-week-old pigs. At 4-week, the tooth eruption is slowed down and enamel mineral addition is twice as fast as in 2-week-old pigs. In 16-week-old pigs, the first incisors are both fully formed and erupted as confirmed by the constantly null mineralization rate.

Where ∆ρ is the rate of mineral addition of enamel along the length of the tooth and expressed in mg_HA_/cm^3^ per mm, $\rho \left(x+\delta x\right)$ is the mineral density at the position x+δx and $\rho \left(x+\delta x\right)$ is the mineral density at the position x . The results are calculated for δx = 1 mm and are presented in Figure 7. To account for the variation in eruption speed between teeth, we considered the rate of mineral addition during enamel formation as a function of the distance from the most newly formed enamel at the cervical margin towards the tooth cusp. Our results indicate significant differences between the age groups, though, it is important to note that the illustrated data do not reflect only the rate of mineralization over time. Rather, the measures capture a snapshot of mineral density at a single time point. The metric used here is dependent on the mineralization rate but also on the growth rate and eruption speed of the tooth.

For the full thickness of the enamel layer, the rate of mineral addition averages 9.8 ± 2.2 mg_HA_/cm^3^/mm (Figure 7) and is lower in incisors of 2-week-old pigs compared to the incisors of 4-week-old pig where it is 16.2 ± 3.6 mg_HA_/cm^3^/mm near the cervical margin. The rate of mineral addition progressively decreases along the length of the tooth.

### Fast formation still produces hard enough enamel

**FIGURE 8:**
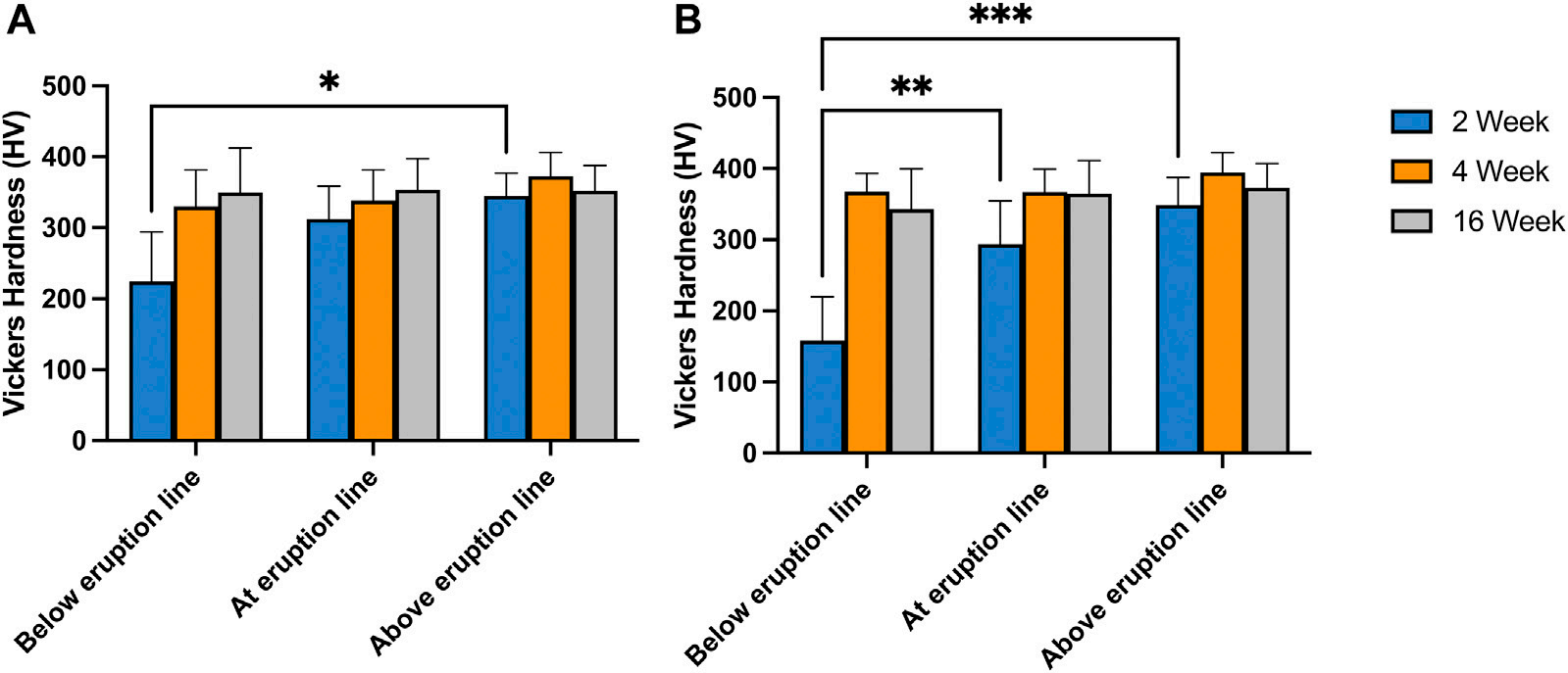
Vickers Hardness measured at various position along the length of first incisors taken from 2-, 4- and 16-week-old pigs. Panel **(A)**. presents the average hardness of enamel measured over a cross-section taken 2 mm below, at and 2 mm above gum line. In panel **(B)** only the results from tests performed 30 µm from the surface of the tooth are presented. The results indicate no significant difference between the hardness of the various locations probes for the incisors of 4- and 16-week-old pigs. In incisors from 2-week-old pigs, the hardness of enamel below gum line is significantly lower than the hardness above eruption line (*p* = .02) **(A)**. The surface microhardness of the same pigs below eruption line are significantly lower than the values at eruption line (*p* = .005) and above eruption line (*p* < .001) **(B)**.

The average microhardness values across the entire thickness of enamel ranged from 224 HV (2-week-old pre-eruptive, SD = 69) to 372 HV (4-week-old post-eruptive SD = 33) among all age groups and locations. There was a significant effect of age, F(2, 15) = 3.73, *p* = 0.049, indicating that the samples became harder with increasing age of individual. However, the effect of location across the entire enamel thickness was not significant, F(2, 15) = 2.58, *p* > 0.05, nor was the association between age and location, F(4, 15) = 1.20, *p* > 0.05 (Figure 8A). A *post hoc* Tukey multiple comparisons test showed only significant difference between the microhardness values between the pre-eruptive (224 HV, SD = 69) and post-eruptive (344 HV, SD = 31) values of the 2-week-old pigs, *p* = 0.02. These results were consistent with the mineral density data derived from microCT measures of the same teeth.

In contrast, the microhardness for surface enamel, specifically for values within 30 µm of the enamel surface, showed a significant positive association with age, F(2, 15) = 15.38, *p* < 0.001, indicating increasing enamel hardness with increasing age of the animal. Additionally, surface hardness was associated with the location relative from the cervical margin along the long axis of the tooth crown, F(2, 15) = 6.86, *p* < 0.01, indicating increasing hardness from apical to incisal. These effects were qualified by a significant interaction of age and location, F(4, 15) = 3.53, *p* = 0.03, reflecting the greater effect of location in the youngest animals. Post-hoc Tukey tests for the surface measurements also indicated that the pre-eruptive microhardness of the 2-week-old pigs was significantly less than the hardness at-eruption (*p* = 0.005), and the post-eruptive hardness (*p* < 0.001) (Figure 8B).

## Discussion

The extremely fast growth rate and early timing of tooth eruption in pigs, especially in breeds selected for farming as used in this study, is at odds with the time required to complete enamel maturation and hardening. As a result, porcine teeth erupt with enamel that is not fully matured. Although it is known that the secretion rate of enamel matrix, that is ameloblast production, is quite variable and can increase if eruption is accelerated (Reid and Dean, 2006; Nacarino-Meneses et al., 2017; An et al., 2018), the speed of enamel maturation does not increase (Robinson et al., 1988a). Our results confirm this for porcine teeth. The enamel portion of the first incisors that is erupted at birth is hypomineralized and only 60% (by weight) mineral in 2-weeks-old pigs. The incomplete mineralization is reflected in significantly lower hardness compared to fully mature enamel of the same teeth at a later time point, as well as in comparison to human teeth.

The low mineral content is accompanied by a higher porosity of the enamel microstructure as observed by electron microscopy. The incisor enamel of 2-week-old animals presents a striking level of porosity seen as channel structures that extend form the crown surface of the tooth through the enamel layer towards the dentin enamel junction. These channels are present even after eruption, suggesting the possibility for continued exchange, facilitated by microstructural organization, for a post-eruptive mineralization process. The surface of 2-week-old enamel also presents some roughness in SEM images suggesting that the surface is not fully formed. While few channels are still present in the enamel microstructure of 4-week-old incisors, they are located in the portion of the tooth crown that is not yet erupted. The enamel crown surface of the erupted portion appears at four weeks after birth sealed and smoother. The enamel near the surface is visibly denser. This change in microstructural appearance is in agreement with the progression of mineral density measured in microCT analyses. Furthermore, the apparent change in enamel porosity over time is supported by our measures of fluorescein penetration. The penetration and attachment of fluorescein into deeper enamel layers is much higher in recently erupted teeth. In contrast, teeth that have been in the oral cavity for an extended period have a sealed crown surface and no fluorescein penetration and related fluorescence is detected in the enamel layer.

It is evident that enamel that is incompletely mineralized at the time of eruption continues to mature in the oral cavity and undergoes substantial changes that likely involve both protein removal and mineral uptake to achieve mineralization and hardening. While the erupted enamel in the incisors of 2-week-old pigs is only partially mineralized, the enamel from the same tooth is in 4-week-old pigs fully mineralized. Accordingly, for the same teeth, the enamel hardness increases within two weeks particularly at the crown surface. Organic material is abundant in enamel before eruption in the incisors at two weeks and four weeks after birth, as seen in SEM images. Fluorescein labelling clearly indicates proteins in enamel up to five weeks after eruption, although the protein is not clearly discernable in SEM images of incisor enamel at four weeks. Interestingly, no fluorescein label is detected in enamel at 11 weeks after eruption, suggesting that mechanisms of protein removal must occur while teeth are in the oral cavity.

Our data suggests that mineralization after eruption occurs fast and leads to substantial increases of mineral density and hardness of the tooth crown within the first weeks of the animals’ life. Indeed, the erupted enamel of 4-week-old pigs appears fully mature with optimal mineral density, hardness, and a microstructure similar to 16-week-old animals. While the incisal part of the incisor forms rapidly, the remaining portion of the tooth forms at a lower rate.

Although we did not directly measure the tooth crown extension rate, we computed the progression of mineral density as a function of distance from the cervical margin. The computational approach assumes that adjacent cross-sections along the long axis of a tooth follow a similar mineralization pattern. Therefore, the difference in mineral density in two adjacent cross-sections, the cervical-incisal mineral addition rate, is inversely related to the time dependent growth rate of the tooth. If the true rate of mineral maturation seen as increase of mineral content over time is constant, then it follows that the larger the difference in mineral density between two adjacent cross-sections, the more time passed for mineral to form and complete enamel maturation and the crown formation. In 4-week-old incisors, the cervical-incisal mineral addition rate measured here reaches values higher than in incisors taken from 2-week-old animals. Consequently, the incisor grows at a slower rate at 4-week when compared to 2-week, and mineral maturation is favored over early eruption of immature enamel and its posteruptive hardening. Observations that are consistent with this concept have been reported in human teeth where differences in crown formation rates are associated with the eruption sequence (Mahoney, 2015). For example, primary teeth that erupt first, such as incisors, have much faster prenatal enamel matrix deposition compared to teeth that erupt later, such as molars. When the enamel growth rate decreases, maturation of enamel prior to its eruption takes priority.

It has been suggested that human enamel is not fully mineralized at the time of eruption (Fejerskov et al., 1984) and that enamel maturation and hardening continue in the oral cavity (Yonan and Fosdick, 1964; Wöltgens et al., 1990; Palti et al., 2008; Lynch, 2013). While human teeth have been shown to be most susceptible to caries during the first years after eruption (Carlos and Gittelsohn, 1965), no data is available for the first few weeks after eruption. Post eruptive maturation has also been reported in rodents (Yonan et al., 1966). Rapid post-eruptive mineralization reported here for pigs might also be present in other species, including humans, despite the differences in enamel microstructure between porcine and human enamel. We have shown that porcine enamel appears to have a microstructure that facilitates exchange processes through channel-like structures that run from the crown surface into deeper enamel layers. The importance of surface porosity for exchange processes with the oral cavity and posteruptive mineralization have been long recognized. For example, it was shown that the permeability of human surface enamel, measured by potassium iodide uptake and back diffusion, decreases significantly with the time maxillary incisors of the permanent dentition are exposed to the oral cavity (Brudevold et al., 1982).

It is well known that enamel maturation and hardening is accompanied by the decrease in protein content. We have shown that the abundance of fluorescein, which binds to proteins, decreases in enamel with increasing time after eruption. This observation is based on two factors. One is the diminished penetration of fluorescein into enamel, determined by the decrease in porosity and surface permeability. The second factor is the amount of protein present in enamel. While we can appreciate the difference in microstructure between 2- and 4-week-old surface enamel, it is unresolved how the decrease in protein content is achieved. The molecular mechanism allowing for fast post-eruptive hardening of enamel remains to be analyzed in future studies.

## Conclusion

Our study highlights that porcine enamel of mandibular incisors that are erupted at birth, can harden extremely fast, within two weeks after birth. This hardening process occurs mainly at the crown surface. The microstructure of surface enamel shows high porosity in incisors from two-week-old piglets and is decreased within 2 weeks to create a densely mineralized surface layer that seals the tooth crown in four-week-old animals. This strategy allows teeth that formed extremely fast and are hypomineralized and soft at birth to achieve the required hardness for an omnivorous diet by weaning age. The short time frame of these drastic changes in microstructure and mineral content has not previously been considered for human enamel maturation.

## Data Availability

The raw data supporting the conclusions of this article will be made available by the authors, without undue reservation.
